# Clinical Characteristics and Outcomes Associated With Oral Anticoagulant Use Among Patients Hospitalized With Intracerebral Hemorrhage

**DOI:** 10.1001/jamanetworkopen.2020.37438

**Published:** 2021-02-16

**Authors:** Ying Xian, Shuaiqi Zhang, Taku Inohara, Maria Grau-Sepulveda, Roland A. Matsouaka, Eric D. Peterson, Jonathan P. Piccini, Eric E. Smith, Kevin N. Sheth, Deepak L. Bhatt, Gregg C. Fonarow, Lee H. Schwamm

**Affiliations:** 1 The Duke Clinical Research Institute, Duke University Medical Center, Durham, North Carolina; 2 Department of Neurology, Duke University Medical Center, Durham, North Carolina; 3 Department of Cardiology, Keio University School of Medicine, Tokyo, Japan; 4 Division of Cardiology, Vancouver General Hospital, University of British Columbia, Vancouver, Canada; 5 Department of Biostatistics and Bioinformatics, Duke University, Durham, North Carolina; 6 Department of Clinical Neurosciences, Hotchkiss Brain Institute, University of Calgary, Calgary, Canada; 7 Department of Neurology, Yale School of Medicine, New Haven, Connecticut; 8 Brigham and Women’s Hospital Heart and Vascular Center, Harvard Medical School, Boston, Massachusetts; 9 Division of Cardiology, University of California at Los Angeles, Los Angeles; 10 Department of Neurology, Massachusetts General Hospital, Boston

## Abstract

**Importance:**

Although the use of factor Xa (FXa) inhibitors has increased substantially over the past decade, there are limited data on characteristics and outcomes of FXa inhibitor–associated intracerebral hemorrhage (ICH).

**Objective:**

To investigate the association between prior oral anticoagulant use (FXa inhibitors, warfarin, or none) and in-hospital outcomes among patients with nontraumatic ICH.

**Design, Setting, and Participants:**

This is a cohort study of 219 701 patients with nontraumatic ICH admitted to 1870 hospitals in the Get With The Guidelines–Stroke registry between October 2013 and May 2018. Data analysis was performed in December 2019.

**Exposures:**

Anticoagulation therapy before ICH.

**Main Outcomes and Measures:**

The primary outcome was in-hospital mortality. Secondary outcomes were a composite measure of in-hospital mortality or discharge to hospice, discharge home, independent ambulation, and modified Rankin Scale (mRS) score at discharge.

**Results:**

Of 219 701 patients (mean [SD] age, 68.2 [15.3] years; 104 940 women [47.8%]), 9202 (4.2%) were taking FXa inhibitors, 21 430 (9.8%) were taking warfarin, and 189 069 (86.0%) were not taking any oral anticoagulant before ICH. Patients taking FXa inhibitors or warfarin were older and had higher prevalence of cardiovascular risk factors. Compared with those not taking an oral anticoagulant (42 660 of 189 069 patients [22.6%]), the in-hospital mortality risk was higher for both FXa inhibitors (2487 of 9202 patients [27.0%]; adjusted odds ratio [aOR], 1.27; 95% CI, 1.20-1.34; *P* < .001) and warfarin (7032 of 21 430 patients [32.8%]; aOR, 1.67; 95% CI, 1.60-1.74; *P* < .001). Both FXa inhibitors (3478 of 9202 patients [37.8%]; aOR, 1.19; 95% CI, 1.13-1.26; *P* < .001) and warfarin (9151 of 21 430 patients [42.7%]; aOR, 1.50; 95% CI, 1.44-1.56; *P* < .001) were associated with higher odds of death or discharge to hospice compared with not taking oral anticoagulation (58 022 of 189 069 patients [30.7%]). Although the rates of discharge home, independent ambulation, mRS scores of 0 or 1, and mRS scores of 0 to 2 were numerically lower among patients taking FXa inhibitors, these differences were not significant compared with patients not taking oral anticoagulants. In contrast, patients taking FXa inhibitors were less likely to die (aOR, 0.76; 95% CI, 0.72-0.81; *P* < .001) or to experience death or discharge to hospice (aOR, 0.79; 95% CI, 0.75-0.84; *P* < .001), more likely to be discharged home (aOR, 1.18; 95% CI, 1.10-1.26; *P* < .001), and had better mRS scores at discharge (eg, mRS scores of 0-1: aOR, 1.24; 95% CI, 1.09-1.40; *P* < .001) than those treated with warfarin. Concomitant warfarin and antiplatelet therapy (either single or dual) was associated with worse outcomes compared with taking warfarin alone (eg, in-hospital mortality for dual-antiplatelet agents: aOR, 2.07; 95% CI, 1.72-2.50; *P *< .001). However, such incremental risk was not significant in patients taking FXa inhibitors.

**Conclusions and Relevance:**

In this cohort study, FXa inhibitor–associated ICH was associated with higher risk of mortality or death or discharge to hospice than not taking an oral anticoagulant, but patients taking FXa inhibitors had better outcomes than those with warfarin-related ICH.

## Key Points

**Question:**

What is the association between prior oral anticoagulant use (factor Xa [FXa] inhibitors, warfarin, or none) and in-hospital outcomes among patients with nontraumatic intracerebral hemorrhage (ICH)?

**Findings:**

In this registry-based cohort study of 219 701 patients with ICH, prior use of FXa inhibitors was associated with higher in-hospital mortality vs no prior anticoagulant use, although FXa inhibitors were associated with lower in-hospital mortality than warfarin.

**Meaning:**

These findings suggest that patients with FXa inhibitor–associated ICH have a higher risk of mortality than those not taking an oral anticoagulant but better outcomes than those with warfarin-related ICH.

## Teaser

This cohort study investigates the association of prior oral anticoagulant use with in-hospital outcomes among patients with nontraumatic intracerebral hemorrhage (ICH).

## Introduction

Intracerebral hemorrhage (ICH) is the most feared complication of oral anticoagulant (OAC) therapy and is associated with high mortality and morbidity. Compared with warfarin, factor Xa (FXa) inhibitors, such as apixaban, rivaroxaban, and edoxaban, reduce the risk of ICH by 52%.^1^ Despite their favorable safety profiles, as many as 0.5% of patients taking FXa inhibitors will still experience an intracranial bleeding event with each year of therapy.^2,3,4^ Although the use of FXa inhibitors has increased substantially over the past decade, prior studies^5,6,7,8^ of FXa inhibitor–associated ICH are limited in size and scope. We have characterized ICH in patients taking non–vitamin K antagonist OACs, including direct thrombin inhibitor (dabigatran) and FXa inhibitors as a group.^9^ Given their differences in mechanism of action and targets at the coagulation cascade, there remains limited experience with FXa inhibitor–associated ICH. In addition, many patients were taking both anticoagulant and antiplatelet therapy before ICH, but the potential incremental risk associated with concomitant therapy remains unknown.

Using data from the American Heart Association and American Stroke Association Get With The Guidelines–Stroke (GWTG-Stroke) registry, we sought to evaluate the characteristics of patients who experienced a nontraumatic ICH with preceding use of FXa inhibitors compared with no OAC or with warfarin, and to determine the risk of mortality and disability according to the type of anticoagulants, and any incremental risk associated with concomitant antiplatelet therapy in nationwide clinical practice.

## Methods

### Data Source

The GWTG-Stroke program is an ongoing, voluntary, national stroke registry and quality improvement initiative sponsored by the American Heart Association and American Stroke Association. Details of GWTG-Stroke registry data collection and variable definitions have been described elsewhere.^10^ Standardized data collection includes patient demographic characteristics, medical history, medications taken before admission, diagnostic testing, brain imaging, in-hospital treatment, and outcomes. The validity and reliability of data collection have been reported in previous research.^11^ Each participating hospital received either human research approval to enroll patients without individual patient consent under the Common Rule (45 CFR §46) or a waiver of authorization and exemption from subsequent review by their institutional review board. IQVIA, Inc serves as the data collection and coordination center. The Duke Clinical Research Institute serves as the data analysis center and has an agreement to analyze the aggregate deidentified data for research purposes. This study was approved by the institutional review board of Duke University. This report follows the Strengthening the Reporting of Observational Studies in Epidemiology (STROBE) reporting guideline for cohort studies.

### Study Population and Variable of Interest

This is a registry-based cohort study of patients hospitalized for nontraumatic ICH in GWTG-Stroke hospitals between October 2013 and May 2018. For the purpose of our analysis, patients with subarachnoid hemorrhage, subdural hematoma, or those taking dabigatran (direct thrombin inhibitor) were not included in the study population. Because andexanet-α, a specific reversal agent for rivaroxaban and apixaban, may affect outcomes in ICH and because the GWTG-Stroke registry did not previously collect the name of the reversal agent, we limited our study period until May 3, 2018, before the US Food and Drug Administration’s approval of andexanet-α. Prior use of OACs was defined as documentation of patients taking an OAC within 7 days before hospital arrival. On the basis of this information, we categorized patients into 3 mutually exclusive groups: direct FXa inhibitors, including rivaroxaban, apixaban, or edoxaban; warfarin; and no OAC use before ICH. The Figure shows details of inclusion and exclusion criteria. Briefly, we excluded patients who had in-hospital stroke onset, transferred out to another hospital, had discharge information missing, left against medical advice, had a prosthetic heart valve, or took anticoagulants other than warfarin or FXa inhibitors before their stroke. After these exclusions, the final study population consisted of 219 701 patients with nontraumatic ICH from 1870 GWTG-Stroke hospitals in the US. Because the National Institute of Health Stroke Scale (NIHSS) score (range, 0-42, with a higher score indicating greater stroke severity) is a critical factor associated with outcomes, we further created a subgroup of individuals with complete NIHSS data (143 340 patients [65.2%]) for a sensitivity analysis.

**Figure.**
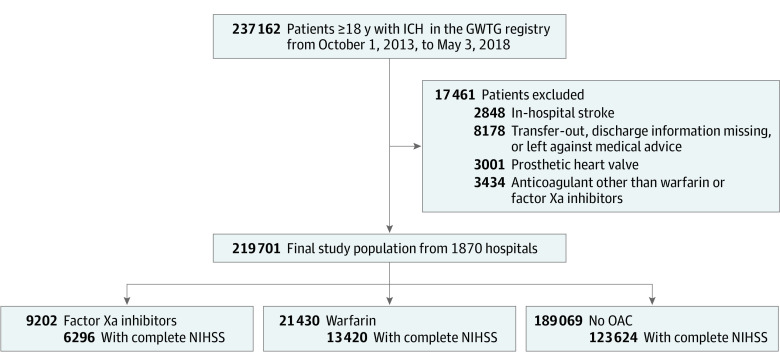
Study Population Flowchart GWTG indicates Get With The Guidelines–Stroke; ICH, intracerebral hemorrhage; NIHSS, National Institute of Health Stroke Scale; OAC, oral anticoagulant.

The primary outcome was in-hospital mortality (yes or no). Secondary outcomes included a composite measure of in-hospital mortality or discharge to hospice (yes or no), discharge disposition (home vs other), ambulatory status at discharge (able to ambulate independently vs not), and modified Rankin Scale (mRS) score at discharge (range, 0 [no symptoms] to 6 [death]). Patients with an mRS score of 0 or 1 were classified as free from substantial disability, and those with an mRS score of 0 to 2 were classified as functionally independent.

### Statistical Analysis

Medians (25th to 75th percentiles) and percentages were used to describe the distribution of continuous and categorical variables, respectively. Baseline characteristics were compared across the 3 prior anticoagulation treatment groups using the Pearson χ^2^ test for categorical variables and Kruskal-Wallis test for continuous variables. Multivariable logistic regression models were performed to investigate the associations of prior anticoagulation therapies with each clinical outcome. Because it is inappropriate to impute outcome measures, complete case analyses were performed for death (100% of cases), discharge disposition (100% of cases), ambulatory status (67.6% of cases), and mRS (67.7% of cases) models.

The prior anticoagulation treatment was included as an independent variable, with no OAC or warfarin as the reference groups. A generalized estimation equations modeling approach was used to account for within-hospital clustering of patients. These analyses adjusted for baseline patient demographic and clinical factors before the index event and hospital characteristics that are expected to be associated with outcome and have been used in prior GWTG-Stroke analyses.^9,12^ Covariates included age, sex, race/ethnicity (admission staff, medical staff, or both recorded the patient’s self-reported race/ethnicity, usually during the registration), insurance, medical history (atrial fibrillation or flutter, coronary artery disease or prior myocardial infarction, prior stroke, prior transient ischemic attack, carotid stenosis, heart failure, hypertension, peripheral vascular disease, diabetes, dyslipidemia, obesity, renal insufficiency, smoking status, and drug or alcohol abuse), arrival and admission information (emergency medical services arrival vs private transportation, transfer-in, and arrived off-hours), medication taken before admission (antihypertensive, lipid-lowering medication, diabetic medication, single-antiplatelet agent [aspirin, clopidogrel, prasugrel, ticagrelor, or ticlopidine], dual-antiplatelet therapy [aspirin plus dipyridamole or aspirin plus clopidogrel, prasugrel, ticagrelor, or ticlopidine], other antiplatelet or combination), rural hospital, hospital number of beds, academic center, geographic region, primary stroke center, and comprehensive stroke center.

NIHSS score was not included in the primary analysis because NIHSS score may lie somewhere between OAC and outcomes, reflecting more rapid hematoma growth before hospital arrival. Instead, the same analyses were replicated in patients with documented NIHSS scores while adjusting for NIHSS score along with other original covariates (143 340 patients [65.2%]) in the sensitivity analysis. The Glasgow Coma Scale (GCS) and ICH scores are commonly used risk stratification scales for ICH. Use of anticoagulation reversal treatment during the hospitalization may have been associated with outcomes. However, these data elements are available for only 86 800 patients (39.5%) hospitalized in centers that opt to report comprehensive stroke center data element. These variables have high missing rates (GCS score, 29.1%; ICH score, 38.1%; and reversal treatment, 72.2%). Therefore, these data were provided for information purpose only and were not included in the risk-adjustment model.

To evaluate the incremental risk of mortality and disability with the concomitant prior antiplatelet therapy, separate multivariable logistic regression models with generalized estimation equations were performed in each OAC group (FXa inhibitors, warfarin, and no OAC), respectively, with antiplatelet treatment category (no antiplatelet, single-antiplatelet agent, or dual-antiplatelet agents) as the independent variable. Patients who were not in any of these 3 antiplatelet groups were excluded from the analysis.

All statistical analyses were performed using SAS statistical software version 9.4 (SAS Institute). All *P* values are 2-sided, with *P* < .05 considered significant. The original analyses were performed in December 2019.

## Results

Baseline characteristics specific to anticoagulants used are shown in Table 1. Of 219 701 patients with nontraumatic ICH (mean [SD] age, 68.2 [15.3] years; 104 940 women [47.8%]), 9202 patients (4.2%) were receiving FXa inhibitors, 21 430 (9.8%) were receiving warfarin, and 189 069 (86.0%) were not receiving any OAC before ICH. Compared with patients without OAC use, patients with prior FXa inhibitor or warfarin use were more likely to be older and non-Hispanic White and to have Medicare insurance; had a higher prevalence of cardiovascular risk factors, including atrial fibrillation or flutter, prior stroke, prior transient ischemic attack, coronary artery disease, peripheral vascular disease, diabetes, hypertension, heart failure, dyslipidemia, and obesity; and had lower a prevalence of tobacco and drug or alcohol abuse (Table 1).

**Table 1.** Baseline Characteristics of Patients and Hospitals by Use of Anticoagulants Before Intracerebral Hemorrhage

Characteristics	Patients or hospitals, No. (%)			*P* value
	Factor Xa inhibitors (n = 9202)	Warfarin (n = 21 430)	No OAC (n = 189 069)	
Patient characteristics				
Age, median (IQR), y	77 (70-84)	77 (69-84)	68 (56-79)	<.001
Women	4510 (49.0)	9928 (46.3)	90 502 (47.9)	<.001
Race/ethnicity				
Non-Hispanic				<.001
White	7229 (78.6)	16 480 (76.9)	112 656 (59.6)	
Black	906 (9.9)	2303 (10.8)	36 783 (19.5)	
Hispanic	422 (4.6)	997 (4.7)	18 292 (9.7)	
Asian	298 (3.2)	771 (3.6)	10 039 (5.3)	
Other	347 (3.8)	879 (4.1)	11 299 (6.0)	
Insurance				
Medicare	4844 (52.6)	11 236 (52.4)	73 106 (38.7)	<.001
Medicaid	629 (6.8)	1569 (7.3)	21 896 (11.6)	
Private insurance	3396 (36.9)	7752 (36.2)	66 084 (35.0)	
Self-pay	96 (1.0)	278 (1.3)	11 725 (6.2)	
Not documented	237 (2.6)	595 (2.8)	16 258 (8.6)	
Medical history				
Atrial fibrillation or flutter	6849 (74.4)	14 317 (66.8)	13 109 (6.9)	<.001
Previous stroke	2872 (31.2)	6106 (28.5)	37 585 (19.9)	<.001
Previous transient ischemic attack	841 (9.1)	1680 (7.8)	8329 (4.4)	<.001
Coronary artery disease or myocardial infarction	2772 (30.1)	6760 (31.5)	27 103 (14.3)	<.001
Carotid stenosis	240 (2.6)	513 (2.4)	2771 (1.5)	<.001
Peripheral vascular disease	510 (5.5)	1502 (7.0)	4406 (2.3)	<.001
Hypertension	7620 (82.8)	17 354 (81.0)	135 672 (71.8)	<.001
Heart failure	1441 (15.7)	4033 (18.8)	9404 (5.0)	<.001
Diabetes	2839 (30.9)	7189 (33.6)	46 764 (24.7)	<.001
Dyslipidemia	4515 (49.1)	10 287 (48.0)	60 118 (31.8)	<.001
Obesity or overweight	2000 (21.7)	4783 (22.3)	34 489 (18.2)	<.001
Renal insufficiency	858 (9.3)	2777 (13.0)	13 713 (7.3)	<.001
Smoker	634 (6.9)	1488 (6.9)	26 560 (14.1)	<.001
Drug or alcohol abuse	331 (3.6)	723 (3.4)	19 001 (10.1)	<.001
Arrival and admission information				
Arrival by emergency medical services	4628 (50.3)	10 228 (47.7)	87 576 (46.3)	<.001
Arrived off-hours^a^	5150 (56.0)	12 176 (56.8)	108 624 (57.5)	.005
Transfer-in	3318 (36.1)	8281 (38.6)	70 962 (37.5)	<.001
Time from symptom onset to arrival, median (IQR), min	210 (75-484)	232 (85-525)	202 (71-494)	<.001
NIHSS score at presentation^b^				
Mean (SD)	11.9 (10.7)	12.5 (11.3)	11.9 (10.8)	<.001
Median (IQR)	9 (3-20)	9 (3-21)	9 (3-20)	
First GCS score^c^				
Mean (SD)	11.4 (4.3)	11.1 (4.5)	11.3 (4.4)	.02
Median (IQR)	14 (8-15)	14 (7-15)	14 (7-15)	
Initial ICH score^d^				
Mean (SD)	1.9 (1.4)	2.0 (1.4)	1.7 (1.4)	<.001
Median (IQR)	2 (1-3)	2 (1-3)	1 (1-3)	
Medication before admission				
Aspirin only	2492 (27.1)	6445 (30.1)	46 974 (24.8)	<.001
Clopidogrel only	242 (2.6)	468 (2.2)	4829 (2.6)	.004
Dual antiplatelet	198 (2.2)	524 (2.5)	8244 (4.4)	<.001
Other antiplatelet	13 (0.2)	54 (0.2)	919 (0.5)	<.001
Antihypertensive	6467 (70.3)	14 820 (69.2)	80 810 (42.7)	<.001
Cholesterol reducer	5203 (56.5)	12 012 (56.1)	59 690 (31.6)	<.001
Diabetic medications	1784 (19.4)	4504 (21.0)	24 783 (13.1)	<.001
Vital signs and laboratory values on admission				
Heart rate, median (IQR), beats/min	80 (69-93)	80 (70-94)	81 (70-94)	<.001
Blood pressure, median (IQR), mm Hg^e^				
Systolic	158 (138-182)	157 (137-180)	160 (140-186)	<.001
Diastolic	86 (73-101)	85 (73-98)	87 (74-103)	<.001
International normalized ratio^f^				
Mean (SD)	1.3 (0.5)	2.6 (1.6)	1.1 (0.5)	<.001
Median (IQR)	1.2 (1.1-1.4)	2.2 (1.54-2.9)	1.0 (1.0-1.1)	
Creatinine clearance,^g^ median (IQR), mL/min/1.73 m^2^	52 (39-70)	51 (37-69)	63 (45-84)	<.001
Use of procoagulant reversal agent^h^	910 (53.4)	3376 (69.1)	1623 (9.2)	<.001
Hospital characteristics				
Beds, median (IQR), No.	457 (310-692)	454 (302-679)	464 (313-692)	<.001
Academic center	7610 (82.7)	18 156 (84.7)	160 907 (85.1)	<.001
Primary stroke center	4784 (52.0)	11 222 (52.4)	100 018 (52.9)	<.001
Comprehensive stroke center	2114 (23.0)	5339 (24.9)	45 489 (24.1)	<.001
Rural hospital	182 (2.0)	481 (2.24)	3618 (1.9)	.004
Region				
West	1509 (16.4)	4206 (19.6)	40 859 (21.6)	<.001
South	3786 (41.1)	6783 (31.7)	75 186 (40.0)	
Midwest	1773 (19.3)	5088 (23.7)	35 735 (18.9)	
Northeast	2134 (23.2)	5353 (25.0)	37 289 (19.7)	

Abbreviations: GCS, Glasgow Coma Scale; ICH, intracerebral hemorrhage; IQR, interquartile range; NIHSS, National Institute of Health Stroke Scale; OAC, oral anticoagulant.

^a^ Refers to arrival at the hospital on weekdays from 6:00 pm to 7:00 am, weekends, or holidays.

^b^ NIHSS score was missing for 76 361 patients (34.8%). NIHSS score ranges from 0 to 42 and a higher score indicates greater stroke severity.

^c^ GCS score was missing for 25 267 of 86 800 patients (29.1%) from hospitals reporting comprehensive stroke data element. The GCS ranges from 3 to 15, and a lower score indicates severe brain injury.

^d^ ICH score was missing for 33 083 of 86 800 patients (38.1%) from hospitals reporting comprehensive stroke data element. ICH score is a clinical grading scale for risk stratification in ICH. The ICH score ranges from 0 to 6, and a higher score indicates greater risk of death.

^e^ Systolic blood pressure was missing for 77 990 patients (35.1%). Diastolic blood pressure was missing for 74 984 patients (34.1%).

^f^ International normalized ratio was missing for 74 338 patients (33.8%).

^g^ Creatinine clearance was missing for 104 778 patients (47.7%).

^h^ Procoagulant reversal agent was missing for 62 626 of 86 800 patients (72.2%) from hospitals reporting comprehensive stroke data element.

Overall, approximately one-third of patients were taking some form of antiplatelet therapy before ICH. Patients taking FXa inhibitors or warfarin were more likely to have concomitant aspirin use than those not taking OAC (2492 [27.1%] vs 6445 [30.1%] vs 46 974 [24.8%]) (Table 1). Patients in all 3 groups had a median initial NIHSS score of 9, whereas patients in the warfarin group had higher mean NIHSS scores (mean [SD], 12.5 [11.3]) than patients taking FXa inhibitors (mean [SD], 11.9 [10.7]) and those not taking OACs (mean [SD], 11.9 [10.8]).

Among patients with documented GCS (61 553 patients) or ICH (53 717 patients) scores, the median GCS score was 14 in all 3 groups, and the median ICH scores were 2 for those taking FXa inhibitors (mean [SD], 1.9 [1.4]) and warfarin (mean [SD], 2.0 [1.4]) and 1 for those not taking OAC (mean [SD], 1.7 [1.4]) (Table 1). Despite the high missing rates and the lack of the name of the reversal agent, 53.4% (910 of 1703 patients excluding 2500 missing) of patients taking FXa inhibitors, 69.1% (3376 of 4889 patients excluding 3164 missing) taking warfarin, and 9.2% (1623 of 17 582 patients excluding 56 962 missing) not taking OAC were treated with some form of reversal or replacement agent during the hospitalization.

### Prior Anticoagulation Treatment and In-hospital Outcomes

The unadjusted in-hospital mortality rates were 22.6% (42 660 of 189 069 patients) for patients not taking OAC, 27.0% (2487 of 9202 patients) for those taking FXa inhibitors, and 32.8% (7032 of 21 430 patients) for those taking warfarin. After risk adjustment, both FXa inhibitors (adjusted odds ratio [aOR], 1.27; 95% CI, 1.20-1.34; *P* < .001) and warfarin (aOR, 1.67; 95% CI, 1.60-1.74; *P* < .001) were associated with greater odds of in-hospital mortality compared with no OAC (Table 2). Similarly, both FXa inhibitors (3478 of 9202 patients [37.8%]; aOR, 1.19; 95% CI, 1.13-1.26; *P* < .001) and warfarin (9151 of 21 430 patients [42.7%]; aOR, 1.50; 95% CI, 1.44-1.56; *P* < .001) were associated with greater odds of death or discharge to hospice than no OAC (58 022 of 189 069 patients [30.7%]). Although the rates of discharge home, independent ambulation, freedom from substantial disability (mRS score 0-1), and functional independence (mRS score 0-2) were numerically lower among patients taking FXa inhibitors, these differences were not significant compared with those not taking OACs. Similar findings were found in the sensitivity analyses after further adjustment with NIHSS score in the subgroup of 143 340 patients with documented NIHSS score at admission (eTable in the Supplement).

**Table 2.** Outcomes by Use of Anticoagulant Prior to Intracerebral Hemorrhage

Outcomes and anticoagulant	Event rate, No./total No. (%)	Adjusted OR (95% CI)^a^^,^^b^	*P* value	Adjusted OR (95% CI)^a^^,^^c^	*P* value
In-hospital mortality					
Factor Xa inhibitors	2487/9202 (27.0)	1.27 (1.20-1.34)	<.001	0.76 (0.72-0.81)	<.001
Warfarin	7032/21 430 (32.8)	1.67 (1.60-1.74)	<.001	1 [Reference]	NA
No OAC	42 660/189 069 (22.6)	1 [Reference]	NA	0.60 (0.57-0.62)	<.001
Death or discharge to hospice					
Factor Xa inhibitors	3478/9202 (37.8)	1.19 (1.13-1.26)	<.001	0.79 (0.75-0.84)	<.001
Warfarin	9151/21 430 (42.7)	1.50 (1.44-1.56)	<.001	1 [Reference]	NA
No OAC	58 022/189 069 (30.7)	1 [Reference]	NA	0.67 (0.64-0.70)	<.001
Discharge home					
Factor Xa inhibitors	1685/9202 (18.3)	0.95 (0.89-1.01)	.12	1.18 (1.10-1.26)	<.001
Warfarin	3491/21 430 (16.3)	0.81 (0.76-0.85)	<.001	1 [Reference]	NA
No OAC	48 893/189 069 (25.9)	1 [Reference]	NA	1.24 (1.18-1.31)	<.001
Independent ambulation at discharge					
Factor Xa inhibitors	1745/6259 (27.9)	0.99 (0.93-1.07)	.87	1.06 (0.98-1.14)	.14
Warfarin	3664/13 256 (27.6)	0.94 (0.89-0.99)	<.001	1 [Reference]	NA
No OAC	45 027/129 036 (34.9)	1 [Reference]	NA	1.07 (1.01-1.12)	.02
Modified Rankin Scale score 0-1					
Factor Xa inhibitors	466/6687 (7.0)	0.95 (0.85-1.07)	.40	1.24 (1.09-1.40)	<.001
Warfarin	906/15 720 (5.8)	0.77 (0.70-0.84)	<.001	1 [Reference]	NA
No OAC	13 043/126 239 (10.3)	1 [Reference]	NA	1.30 (1.19-1.42)	<.001
Modified Rankin Scale score 0-2					
Factor Xa inhibitors	724/6687 (10.8)	0.95 (0.86-1.05)	.29	1.25 (1.13-1.39)	<.001
Warfarin	1420/15 720 (9.0)	0.76 (0.70-0.82)	<.001	1 [Reference]	NA
No OAC	19 346/126 239 (15.3)	1 [Reference]	NA	1.32 (1.22-1.42)	<.001

Abbreviations: NA, not applicable; OAC, oral anticoagulant; OR, odds ratio.

^a^ Adjusted for age, sex, race/ethnicity, insurance, medical history (atrial fibrillation or flutter, coronary artery disease or prior myocardial infarction, prior stroke, prior transient ischemic attack, carotid stenosis, heart failure, hypertension, peripheral vascular disease, diabetes, dyslipidemia, obesity, renal insufficiency, smoking status, and drug or alcohol abuse), transport by emergency medical services, transfer in, arrived during off-hours, medication prior to admission (antihypertensive, lipid-lowering medication, diabetic medication, single-antiplatelet agent [aspirin, clopidogrel, prasugrel, ticagrelor, or ticlopidine], dual-antiplatelet therapy [aspirin plus dipyridamole or aspirin plus clopidogrel, prasugrel, ticagrelor, or ticlopidine], other antiplatelet, or combination), rural hospital, hospital number of beds, academic center, geographic regions, primary stroke center, and comprehensive stroke center.

^b^ Reference values are for no OAC.

^c^ Reference values are for warfarin.

Compared with those treated with warfarin, patients receiving FXa inhibitors were less likely to die (aOR, 0.76; 95% CI, 0.72-0.81; *P* < .001) and were less likely to die or be discharged to hospice (aOR, 0.79; 95% CI, 0.75-0.84; *P* < .001) (Table 2). In addition, patients taking FXa inhibitors were more likely to be discharged home (aOR, 1.18; 95% CI, 1.10-1.26; *P* < .001) and have better mRS scores at discharge (aOR, 1.24 [95% CI, 1.09-1.40] for mRS scores of 0-1 and aOR 1.25 [95% CI, 1.13-1.39] for mRS scores of 0-2; *P* < .001 for both) than those taking warfarin. These findings were consistent after further adjustment with NIHSS score.

### Incremental Risk of Mortality and Disability With Concomitant Anticoagulant and Antiplatelet Therapy

Among patients with ICH with prior use of FXa inhibitors, there were no significant incremental risks of mortality or functional outcomes at discharge with either single-antiplatelet or dual-antiplatelet agents compared with patients without concomitant antiplatelet use (Table 3). By contrast, in patients with prior use of warfarin, both single-antiplatelet and dual-antiplatelet agents were associated with higher odds of in-hospital mortality (eg, dual-antiplatelet agent: aOR, 2.07; 95% CI, 1.72-2.50; *P *< .001) and death or discharge to hospice (eg, dual-antiplatelet agent: aOR, 1.86; 95% CI, 1.54-2.26; *P* < .001). In addition, these patients were less likely to be discharged home or have better mRS score at discharge, although some of these differences were not significant. Among patients without prior use of OAC, only dual-antiplatelet agents were associated with worse outcomes at discharge. In contrast, patients taking single-antiplatelet agents were more likely to be discharged to home, ambulate independently, and have mRS scores of 0 to 2 at discharge.

**Table 3.** Incremental Risk of Concomitant Antiplatelet Therapy by the Type of Anticoagulant Prior to Intracerebral Hemorrhage

Outcomes and antiplatelet	Event rate, No./total No. (%)	Adjusted OR (95% CI)^a^	*P* value
Factor Xa inhibitors			
In-hospital mortality			
No antiplatelet agent	1701/6257 (27.2)	1 [Reference]	NA
Single-antiplatelet agent	729/2740 (26.6)	1.07 (0.96-1.19)	.22
Dual-antiplatelet agents	55/198 (27.8)	1.19 (0.86-1.66)	.30
Death or discharge to hospice			
No antiplatelet agent	2392/6257 (38.2)	1 [Reference]	NA
Single-antiplatelet agent	1009/2740 (36.8)	1.06 (0.96-1.18)	.23
Dual-antiplatelet agents	74/198 (37.4)	1.21 (0.89-1.66)	.23
Discharge home			
No antiplatelet agent	1146/6257 (18.3)	1 [Reference]	NA
Single-antiplatelet agent	505/2740 (18.4)	0.95 (0.83-1.09)	.48
Dual-antiplatelet agents	34/198 (17.2)	0.69 (0.45-1.05)	.08
Independent ambulation at discharge			
No antiplatelet agent	1198/4249 (28.2)	1 [Reference]	NA
Single-antiplatelet agent	506/1868 (27.1)	0.89 (0.77-1.03)	.11
Dual-antiplatelet agents	41/137 (29.9)	0.92 (0.63-1.35)	.67
Modified Rankin Scale score 0-1			
No antiplatelet agent	318/4553 (7.0)	1 [Reference]	NA
Single-antiplatelet agent	137/1986 (6.9)	0.85 (0.67-1.08)	.19
Dual-antiplatelet agents	11/144 (7.6)	0.88 (0.48-1.63)	.69
Modified Rankin Scale score 0-2			
No antiplatelet agent	500/4553 (11.0)	1 [Reference]	NA
Single-antiplatelet agent	208/1986 (10.5)	0.82 (0.66-1.01)	.06
Dual-antiplatelet agents	16/144 (11.1)	0.79 (0.47-1.32)	.37
Warfarin			
In-hospital mortality			
No antiplatelet agent	4472/13 966 (32.0)	1 [Reference]	NA
Single-antiplatelet agent	2306/6921 (33.3)	1.16 (1.09-1.24)	<.001
Dual-antiplatelet agents	246/524 (46.9)	2.07 (1.72-2.50)	<.001
Death or discharge to hospice			
No antiplatelet agent	5916/13 966 (42.4)	1 [Reference]	NA
Single-antiplatelet agent	2944/6921 (42.5)	1.13 (1.06-1.21)	<.001
Dual-antiplatelet agents	280/524 (53.4)	1.86 (1.54-2.26)	<.001
Discharge home			
No antiplatelet agent	2285/13 966 (16.4)	1 [Reference]	NA
Single-antiplatelet agent	1134/6921 (16.4)	0.96 (0.88-1.05)	.39
Dual-antiplatelet agents	72/524 (13.7)	0.71 (0.55-0.94)	.01
Independent ambulation at discharge			
No antiplatelet agent	2391/8727 (27.4)	1 [Reference]	NA
Single-antiplatelet agent	1195/4264 (28.0)	1.00 (0.92-1.10)	.91
Dual-antiplatelet agents	76/255 (29.8)	1.04 (0.78-1.38)	.81
Modified Rankin Scale score 0-1			
No antiplatelet agent	599/10 220 (5.9)	1 [Reference]	NA
Single-antiplatelet agent	283/5071 (5.6)	0.81 (0.69-0.96)	.02
Dual-antiplatelet agents	23/412 (5.6)	0.73 (0.45-1.19)	.21
Modified Rankin Scale score 0-2			
No antiplatelet agent	927/10 220 (9.1)	1 [Reference]	NA
Single-antiplatelet agent	459/5071 (9.1)	0.87 (0.75-1.00)	.05
Dual-antiplatelet agents	33/412 (8.0)	0.66 (0.43-1.01)	.05
No OAC			
In-hospital mortality			
No antiplatelet agent	28749/128 754 (22.3)	1 [Reference]	NA
Single-antiplatelet agent	11400/51 874 (22.0)	1.01 (0.98-1.05)	.42
Dual-antiplatelet agents	2466/8244 (29.9)	1.56 (1.47-1.65)	<.001
Death or discharge to hospice			
No antiplatelet agent	38096/128 754 (29.6)	1 [Reference]	NA
Single-antiplatelet agent	16637/51 874 (32.1)	1.00 (0.97-1.03)	.95
Dual-antiplatelet agents	3228/8244 (39.2)	1.38 (1.30-1.46)	<.001
Discharge home			
No antiplatelet agent	35360/128 754 (27.5)	1 [Reference]	NA
Single-antiplatelet agent	11823/51 874 (22.8)	1.04 (1.01-1.07)	.008
Dual-antiplatelet agents	1657/8244 (20.1)	0.91 (0.85-0.97)	.004
Independent ambulation at discharge			
No antiplatelet agent	31591/86 122 (36.7)	1 [Reference]	NA
Single-antiplatelet agent	11782/37 441 (31.5)	1.04 (1.01-1.07)	.02
Dual-antiplatelet agents	1606/5333 (30.1)	1.03 (0.96-1.10)	.40
Modified Rankin Scale score 0-1			
No antiplatelet agent	9445/84 302 (11.2)	1 [Reference]	NA
Single-antiplatelet agent	3163/35801 (8.8)	1.04 (0.98-1.10)	.17
Dual-antiplatelet agents	425/6013 (7.1)	0.86 (0.76-0.97)	.02
Modified Rankin Scale score 0-2			
No antiplatelet agent	13734/84 302 (16.3)	1 [Reference]	NA
Single-antiplatelet agent	4929/35 801 (13.8)	1.07 (1.02-1.13)	.006
Dual-antiplatelet agents	665/6013 (11.1)	0.87 (0.78-0.97)	.01

Abbreviations: NA, not applicable; OAC, oral anticoagulant; OR, odds ratio.

^a^ Adjustment for age, sex, race/ethnicity, insurance, medical history (atrial fibrillation or flutter, coronary artery disease or prior myocardial infarction, prior stroke, prior transient ischemic attack, carotid stenosis, heart failure, hypertension, peripheral vascular disease, diabetes, dyslipidemia, obesity, renal insufficiency, smoking status, and drug or alcohol abuse), transport by emergency medical services, transfer in, arrived during off-hours, antihypertensive, lipid-lowering medication prior to admission, rural hospital, hospital number of beds, academic center, geographic regions, primary stroke center, and comprehensive stroke center.

## Discussion

In this cohort study of more than 200 000 patients with nontraumatic ICH, 4.2% were receiving FXa inhibitors and 9.8% were receiving warfarin before stroke. Although patients receiving FXa inhibitors were more likely to have favorable outcomes than those taking warfarin in terms of mortality and functional outcomes, prior use of FXa inhibitors was associated with increased odds of mortality and death or discharge to hospice compared with those not taking OAC, with more than 1 in 4 patients still dying in the hospital. These findings highlight the need to identify optimal strategies to care for these complex but increasingly common clinical challenges. In addition, concomitant antiplatelet use was common in patients with ICHs associated with use of FXa inhibitors and warfarin. Although both single-antiplatelet and dual-antiplatelet therapy were associated with increased odds of worse outcome among patients taking warfarin, such significant differences were not observed among patients with ICH taking concomitant FXa inhibitors and antiplatelet agents.

The current US and European guidelines recommend FXa inhibitors and direct thrombin inhibitor over warfarin for stroke prevention in high-risk patients with atrial fibrillation.^13,14^ FXa inhibitors do not require therapeutic monitoring and are associated with fewer adverse effects and lower rates of bleeding complications, particularly hemorrhagic stroke, than warfarin. As a result, FXa inhibitors are increasingly used in clinical practice. Nonetheless, the rapid adoption of FXa inhibitors will most likely lead to a further increase of FXa inhibitor–associated ICH, highlighting the need to better understand the characteristics, treatment patterns, and prognosis of these patients. However, such studies are challenging because of the improved safety profile of FXa inhibitors and the lower event rates of FXa inhibitor–associated ICH. For instance, of 30 300 patients randomized to receive rivaroxaban, apixaban, or edoxaban in pivotal FXa inhibitor trials,^2,3,4^ only 141 patients experienced a hemorrhagic stroke.

To our knowledge, this study provides the largest and most comprehensive assessment of FXa inhibitor–associated ICH. Overall, we observed higher risk profiles in terms of age and comorbidities in patients taking FXa inhibitors than spontaneous ICH without preceding use of OAC. Despite no significant differences in the likelihood of discharge home and functional status at discharge, FXa inhibitor–associated ICH was associated with 27% higher odds (aOR, 1.27) of in-hospital death and 19% higher odds (aOR, 1.19) of death or discharge to hospice than those without preceding use of OAC. In contrast, patients with FXa inhibitor–associated ICH had lower odds of death or discharge to hospice and higher odds of discharge home, freedom from substantial disability, and functional independence compared with patients with warfarin-associated ICH. Although the exact reason for better outcomes for FXa inhibitor–associated ICH vs warfarin-associated ICH remains unknown, we observed less severe stroke at presentation as measured by the NIHSS score in patients with FXa inhibitor–associated ICH. Although the differences in stroke severity may be explained by the underlying risk profiles between patients receiving FXa inhibitor and warfarin, previous studies have suggested smaller ICH volume, less hematoma expansion, and fewer concomitant intraventricular hemorrhage in patients with ICH taking non–vitamin K OACs.^8,15,16^ Importantly, in certain clinical scenarios, such as atrial fibrillation with recent percutaneous coronary intervention, patients may be required to take both anticoagulant and antiplatelet agents for secondary prevention. Once bleeding complications occurred, however, we found that patients with warfarin-associated ICH had worse outcomes with both single-antiplatelet and dual-antiplatelet therapy. In contrast, such differences were not observed among patients with concomitant FXa inhibitor and antiplatelet agent. These findings suggest that non–vitamin K OACs may be a better choice than warfarin when combination strategy is warranted.

Unlike warfarin-related ICH, the best management strategy for FXa inhibitor–associated ICH remains uncertain. The 2019 European Stroke Organisation Guidelines^17,18^ recommend andexanet-α in ICH with rivaroxaban or apixaban or prothrombin complex concentrate to normalize coagulation tests if specific reversal agents are not available. However, the quality of evidence is low because of a lack of randomized clinical trials and uncertainty regarding the benefit and harm of the reversal treatment.^17,18^ Comparing the impact of various reversal strategies can be difficult because of selection bias and concomitant medication use in ICH, making it hard to disentangle the effectiveness of various treatments.^19^ Furthermore, because anticoagulant levels are rarely captured, further research is required to measure clinical outcomes in the context of anticoagulant levels in addition to bleeding size and severity. In the subgroup of patients from hospitals reporting comprehensive stroke center data elements, we found that 53.4% of patients with FXa inhibitors received some form of reversal or replacement agent. Despite the high missing rates and no specific information regarding the reversal strategy, this finding suggests that reversal treatment was not uncommon among patients with ICH occurring during use of FXa inhibitors. Because our study was conducted before the approval of andexanet-α in the US, in the absence of head-to-head clinical trials, these data may be used as a historical control for future research to compare the efficacy and safety of andexanet-α vs nonspecific reversal treatment in patients with FXa inhibitor–associated ICH.

### Limitations

There are limitations that should be kept in mind when interpreting these data. First, this was an observational analysis. Treatment selection and residual or unmeasured confounding could affect the validity of study findings and potentially account for the differences in risk-adjusted outcomes. Because treatment was given before the ICH, it is impossible to randomize patients with different OAC regimens. Despite the observational nature, our study still provides important clinical insights to FXa inhibitor–associated ICH, especially given the low incidence rates in pivotal trials. Second, prior use of OAC was defined as documentation of patients taking an OAC within 7 days, yet timing and dose of last OAC intake were not available. It is possible that some patients may have received a lower dose or stopped taking OAC, which consequently influenced ICH outcomes. However, it could be argued that patients are more likely to experience an ischemic stroke rather than an ICH in such scenarios.

Third, some patients were excluded from the ambulatory status or functional outcome models because of missing outcomes. Although this approach may bias the results of this analysis, it is unlikely that physicians will report outcome measures differently according to the type of OAC taken before the stroke. Fourth, ICH volume and location and the presence of hematoma expansion were not available in the registry, which may explain the differences in outcomes between ICH patients with or without OAC use. Fifth, platelet transfusion may have influenced outcomes in patients taking concomitant antiplatelet and anticoagulant therapy. However, such data were not collected in the registry. Sixth, data are obtained from hospitals participating in the GWTG-Stroke program and may not be able to be extrapolated to patients treated in hospitals outside the registry or to patients in other countries. Notwithstanding these limitations, GWTG-Stroke is the largest stroke registry in the US, covering approximately three-fourths of the US population. Furthermore, ICH cases tend to be concentrated at or transferred to tertiary hospitals. Given the higher representation of high-volume, academic, and stroke centers in the GWTG-Stroke registry, the study population of this investigation is potentially more representative of OAC-associated ICH in the US.

## Conclusions

In conclusion, FXa inhibitor–associated ICH remains a devastating complication of OAC therapy, with 27.0% of patients in this study dying in hospital. Although associated with better outcomes than warfarin, this study found that preceding use of FXa inhibitors was associated with higher risk of mortality and death or discharge to hospice than ICH without prior use of OACs. Further study is warranted to identify the best treatment strategies for FXa inhibitor–associated ICH.

## Notes

Supplement. **eTable.** Outcomes by Use of Anticoagulant Prior to Intracerebral Hemorrhage, With Further Adjustment With National Institute of Health Stroke Scale (NIHSS)
