# Supplementary material for: Clinical Characteristics and Outcomes Associated With Oral Anticoagulant Use Among Patients Hospitalized With Intracerebral Hemorrhage
Source: JAMA Netw Open. 2021 Feb 16;4(2):e2037438. doi: 10.1001/jamanetworkopen.2020.37438 (PMC7887660; doi:10.1001/jamanetworkopen.2020.37438)
Supplement: Supplement. — eTable. Outcomes by Use of Anticoagulant Prior to Intracerebral Hemorrhage, With Further Adjustment With National Institute of Health Stroke Scale (NIHSS) [file jamanetwopen-e2037438-s001.pdf]

Supplemental Online Content

Xian Y, Zhang S, Inohara T, et al. Clinical characteristics and outcomes associated with oral anticoagulant use among patients hospitalized with intracerebral hemorrhage. *JAMA Netw Open*. 2021;4(2):e2037438. doi:10.1001/jamanetworkopen.2020.37438

eTable. Outcomes by Use of Anticoagulant Prior to Intracerebral Hemorrhage, With Further Adjustment With National Institute of Health Stroke Scale (NIHSS)

This supplemental material has been provided by the authors to give readers additional information about their work.

© 2021 Xian Y et al. *JAMA Network Open.*

eTable. Outcomes by Use of Anticoagulant Prior to Intracerebral Hemorrhage, With Further Adjustment With National Institute of Health Stroke Scale (NIHSS)

**Outcomes**	**Anticoagulant**	**Event Rate (%)**	**Adjusted OR (95% CI)**	**P value**	**Adjusted OR (95% CI)**	**P value**
In-hospital mortality	Factor Xa Inhibitors	1452/6296 (23.1)	1.38 (1.27-1.51)	<0.001	0.77 (0.71-0.84)	<0.001
	Warfarin	3788/13,420 (28.2)	1.78 (1.68-1.90)	<0.001	Reference	
	No OAC	22,409/123,624 (18.1)	Reference		0.56 (0.53-0.60)	<0.001
Death or discharge to hospice	Factor Xa Inhibitors	2126/6296 (33.8)	1.26 (1.16-1.36)	<0.001	0.80 (0.73-0.86)	<0.001
	Warfarin	5163/13,420 (38.5)	1.58 (1.49-1.68)	<0.001	Reference	
	No OAC	32,167/123,624 (26.0)	Reference		0.63 (0.60-0.67)	<0.001
Discharge home	Factor Xa Inhibitors	1164/6296 (18.5)	0.90 (0.83-0.98)	0.02	1.12 (1.03-1.23)	0.009
	Warfarin	2248/13,420 (16.8)	0.81 (0.76-0.86)	<0.001	Reference	
	No OAC	33,314/123,624 (26.9)	Reference		1.24 (1.16-1.32)	<0.001
Independent ambulation at discharge	Factor Xa Inhibitors	1266/4559 (27.8)	0.93 (0.85-1.02)	0.12	1.05 (0.96-1.16)	0.29
	Warfarin	2519/8986 (28.0)	0.88 (0.83-0.94)	<0.001	Reference	
	No OAC	32,059/91,100 (35.2)	Reference		1.13 (1.06-1.21)	<0.001
Modified Rankin Scale Score 0-1	Factor Xa Inhibitors	356/4578 (7.8)	0.93 (0.80-1.07)	0.32	1.12 (0.96-1.29)	0.15
	Warfarin	671/9899 (6.8)	0.83 (0.75-0.93)	<0.001	Reference	
	No OAC	9679/82,867 (11.7)	Reference		1.20 (1.08-1.34)	<0.001
Modified Rankin Scale Score 0-2	Factor Xa Inhibitors	564/4578 (12.3)	0.95 (0.84-1.07)	0.41	1.21 (1.07-1.38)	0.003
	Warfarin	1028/9899 (10.4)	0.78 (0.71-0.86)	<0.001	Reference	
	No OAC	14449/82,867 (17.4)	Reference		1.28 (1.17-1.40)	<0.001

Abbreviation: 95% CI, 95% confidence interval; OAC, oral anticoagulant; OR, odds ratio.

Same adjustment variables as those in Table 2 plus NIHSS in patients with documented NIHSS at admission (n=143,340)

© 2021 Xian Y et al. *JAMA Network Open.*
